# Genomic epidemiology and resistant genes of Acinetobacter baumannii clinical strains in Vietnamese hospitals

**DOI:** 10.1099/jmm.0.001922

**Published:** 2024-10-30

**Authors:** Vu Nhi Ha, Hoang Tran Huy, Trung Nguyen Đac, Phuong Anh Nguyen, Le Duy Cuong

**Affiliations:** 1 Thai Nguyen University of Medicine and Pharmacy, No. 284 Luong Ngoc Quyen Street, Quang Trung Ward, Thai Nguyen City, Thai Nguyen Province, Vietnam; 2 National Institute of Hygiene and Epidemiology, 1st Yersin, Hanoi city, Vietnam; 3 Department of Experiment Medicine, 108 Military Central Hospital, 1st Tran Hung Dao Street, Bach Dang Ward, Hai Ba Trung District, Hanoi City, Vietnam

**Keywords:** *Acinetobacter baumannii*, bioinformatics, multidrug resistance, Vietnam, whole-genome sequencing

## Abstract

**Introduction.***Acinetobacter baumannii* is a common cause of multidrug-resistant (MDR) nosocomial infections worldwide, including Vietnam.

**Hypothesis.** Analysis of crucial genetic factors may link to epidemiological characteristics and antibiotic resistance of *A. baumannii* clinical strains in Vietnamese hospitals.

**Methodology.** Fifty-one *A*. *baumannii* clinical strains from six different tertiary hospitals in Vietnam were analysed using whole genome sequencing (WGS), between 2017 and 2019.

**Results.** Eleven sequence types (STs) were identified, including four STs reported for the first time in Vietnam based on the PubMLST database and three new STs not previously documented. ST1336, ST1260 and ST575 were found exclusively in Vietnam. These STs were widely distributed in all hospitals in Vietnam, with ST2 and ST571 being the most dominant. Resistant rates to eight antibiotics, belonging to four antibiotic groups, were very high (72.5–94.1 %) with high MIC values, while resistance to colistin was 29.4%. Fifty-one isolates were identified as MDR, with 100% (51/51) isolates carrying antimicrobial-resistant (AMR) genes, and 52 antibiotic-resistant genes were detected among these strains, including β-lactam (22 genes), chloramphenicol (5 genes), lincosamide (2 genes), aminoglycoside (11 genes), rifampicin (1 gene), quinolone (2 genes), sulfonamide and trimethoprim (4 genes) and tetracycline (5 genes) resistance. The most commonly found mobile structures carried partial or complete transposons: ISaba24/ISEc29/ISEc35 contains a series of antibiotic-resistant genes.

**Conclusion.** The WGS results of the 51 strains of *A. baumannii* provided important information regarding the distribution of STs and associated antibiotic-resistant genes among *A. baumannii* strains.

## Introduction

*Acinetobacter baumannii* is an opportunistic Gram-negative bacterium pathogen infecting, especially, immunocompromised patients. Its ability to survive in hostile conditions and high level of intrinsic and acquired antimicrobial resistance (AMR) have contributed to its success as a pathogen in hospital settings [1,3], including in Vietnam [4,5]. *A. baumannii* is an opportunistic pathogen that belongs to the ESKAPE group categorized by the Infectious Diseases Society of America [6]. The World Health Organization has announced this bacterium as a priority 1 critical pathogen since 2017 [7]. Three major global clones of * A. baumannii*, including IC-I, IC-II and IC-III have emerged as high-risk pandemic lineages in hospital settings [8]. The genome plasticity of *A. baumannii* further enhances its ability to adapt and persist in hospital environments, leading to the emergence of multidrug-resistant (MDR) strains on a global scale [9,11]. The bacterium manifests intrinsic resistance to many classes of antimicrobials and develops further ability of resistance to virtually all other classes of agents that are used in clinical practices to treat Gram-negative infections [12,14].

Recently, the sequencing of several *A. baumannii* genomes revealed a wide range of AMR genes, many of which are associated with transposable elements and insertion sequences, and which might be detected in genomic islands such as AbaR [15,17]. Some AbaR islands have been described, which can vary in size and are dynamically reshaped mainly due to the activities of transposases, recombinases and integrases [16,18]. On the other side, resistant genes can also be found within plasmids, which can be exchanged intra- and inter-species [19,20] and even by prophages [21].

In this study, we used whole-genome sequencing (WGS) along with bioinformatics to analyse the entire genomes of 51 clinical strains of *A. baumannii* from six tertiary hospitals situated in three distinct regions of Vietnam: the North, Central and South regions. We then compared these bacterial genomes with a set of control genomes that were previously sequenced in order to assess the epidemiological distribution of sequence type (ST) patterns among clinical strains of *A. baumannii* and to identify crucial genetic factors linked to antibiotic resistance in Vietnamese hospitals.

## Methods

### Study location and sampling

Fifty-one *A*. *baumannii* strains were isolated from clinical samples of inpatients with infectious symptoms and syndromes attending six hospitals in distinct regions of Vietnam from 2017 to 2019, in which Thanh Nhan Hospital, Huu Nghi Hospital, 108 Central Military Hospital and Saint Paul Hospital are in the North, Hue Central Hospital is in the Central region and Can Tho Hospital is in the South region. Patients with incomplete medical records, aged <18 years, or with polymicrobial infections were excluded. The isolates were obtained from different infection sites, including blood (24), pus (4), urine (6), pharyngeal secretion (12) and bronchial secretion (5). The identification procedure for *A. baumannii* was performed separately according to routine techniques at the Department of Microbiology in each hospital.

### Bacterial culture and isolation

The *A. baumannii* strains were cultured onto MacConkey agar, and a specific colony was selected based on phenotypic morphology; after that, this colony was subcultured into Luria–Bertani agar (Invitrogen, USA). The strain was identified by the MALDI-TOF MS machine (Microflex LT instrument using the FlexControl 3.0 and MALDI BioTyper 2.0 software; Bruker Daltonics GmbH, Leipzig, Germany). Finally, the strain was stored at −70 to −80 °C as glycerol stocks by putting four to five colonies into sterilized 1 ml Luria-Bertani agar + 25% glycerol (Invitrogen). The procedure was carried out at the National Institute of Hygiene and Epidemiology, Hanoi, Vietnam.

### AMR phenotyping of the isolates

The MIC test was determined on 51 *A*. *baumannii* isolates to identify susceptible or resistant phenotypes to the medications. *A. baumannii* was tested for nine antibiotic drugs belonging to four different classes, including imipenem, meropenem and doripenem of the carbapenem class; ceftazidime and cefepime of the cephalosporin class; gentamycin and amikacin of the aminoglycosides class and ciprofloxacin and levofloxacin of the fluoroquinolone class. MICs were determined centrally using the agar dilution and microdilution methods for colistin (Sigma-Aldrich) according to the Clinical and Laboratory Standards Institute (2018) and the European Committee on Antimicrobial Susceptibility Testing (2018) [22,23]. *Escherichia coli* ATCC 25922 and *Pseudomonas aeruginosa* ATCC 27853 were used as controls.

### DNA extraction, library preparation and next-generation sequencing

#### DNA extraction and purification

The genomic DNA of the strains was extracted and purified using the QIAamp DNA Mini Kit following the manufacturer’s instructions. The quality and quantity of DNA were determined by gel electrophoresis and the NanoDrop 2000 spectrophotometer (Nano Drop Technologies, Wilmington, DE).

#### Library preparation and sequencing

The input of the purified DNA was taken, and the standard protocol for the Nextera XT DNA Sample Prep Kit (Illumina, San Diego, CA) was used for library construction. Finally, the genome was sequenced by the Illumina MiSeq platform using the MiSeq V3 (600 cycles) reagent kit (Illumina, San Diego, CA).

### Genome assembly, gene prediction and functional annotation

The quality of the raw reads was assessed by FastQC (https://www.bioinformatics.babraham.ac.uk/projects/fastqc/) and pre-processed by Trimmomatic 0.36 [24] (parameters: ‘NexteraPE-PE.fa:2 : 30 : 10’, ‘LEADING:10’, ‘TRAI LING:10’, ‘SLIDINGWINDOW:4 : 20’ and ‘MIN LEN:100’). After filtering, high-quality reads were assembled by SPAdes Genome Assembler 3.13.0 [20], with parameters ‘Coverage Cutoff: Off’ and ‘Automatically choose k-mer values: “True” and ‘Careful correction: True”’. Afterwards, contigs shorter than 1000 base pairs were removed from the final assembly by seqtk seq 1.2 (https://github.com/lh3/seqtk) with the ‘-L’ parameter. The quality of the assembled genome was evaluated using Quast 4.4 [25] and BUSCO [26] with default parameters. Open reading frames and RNA genes of the *A. baumannii* TN81 strain were predicted and functionally annotated by PROKKA v1.14.5 [27]. For the discovery of AMR genes of strain TN81, we screened assembled contig by using the ABRicate tool (https://github.com/tseemann/abricate) through several different databases including ARG-ANNOT [28] NCBI, CARD [29] and ResFinder [30]. The threshold of gene detection was set to 60% identity and 60% coverage as default in the software.

### Phylogenetic tree and multi-locus sequence typing (MLST) analysis

The 16S rRNA gene sequence and core genes from the *A. baumannii* strains were compared with *Acinetobacter baylyi* ADP1 (the same genus), *Acinetobacter junii* lzh-X15 (the same genus) and *P. aeruginosa* DSM 50071 obtained from GenBank to confirm *A. baumannii* species. The Molecular Evolutionary Genetics Analysis [31], version X, was used to examine the evolutionary connections between the 16S rRNA gene sequence and score genes from the *A. baumannii* strains. This software was used by many previous studies to construct the phylogenetic trees [32,34]. All sequences were aligned by muscle [35] with the UPGMA parameter, and then phylogenetic analyses of all *Acinetobacter* isolates were undertaken using neighbour-joining with 1000 bootstrap resampling replicates for each. Finally, the phylogenetic tree was graphically visualized using iTOL [36]. The Sequence ST assignation of *A. baumannii* strains was obtained from the PubMLST database (https://pubmlst.org) [37] by *in silico* approach. The genome FASTA sequences of an assembled contig were uploaded into the database by using the MLST v2.6 script (https://github.com/tseemann/mlst) with the Pasteur scheme on the *A. baumannii* MLST section, which will generate allelic profiles and STs.

### *De novo* plasmid assembly and antibiotic-resistant gene detection

The presence of plasmids in the genomes was identified using the following bioinformatic tools: PlasmidFinder [38], plasmidSPAdes v3.10.1 [39], BlastN on NCBI [40] and Kablammo (http://kablammo.wasmuthlab.org/) [41]. First, PlasmidFinder was used to identify plasmid replicon locations in the assembled genome. To enhance plasmid detection and to detect all contigs representing a specific plasmid, the plasmidSPAdes tool was then utilized. The algorithm in plasmidSPAdes was able to predict which contigs belong to plasmid DNA and to authorize those contigs into components. Each component is recognized as a putative plasmid composed of one or more contigs. Thus, all of these components (putative plasmids and SPAdes output) from the strains were used to seek the most similar/reference plasmids at the NCBI nt database using BlastN. Finally, the sequences of the identified reference plasmids from NCBI were searched back into the strains using Kablammo software. The putative plasmids were also examined for their antibiotic resistance ability through the prediction of AMR genes on the ResFinder database [30] with default parameters.

## Results

### MLST analysis of 16S rRNA and core genes of 51 *A. baumannii* strains

**Fig. 1.**
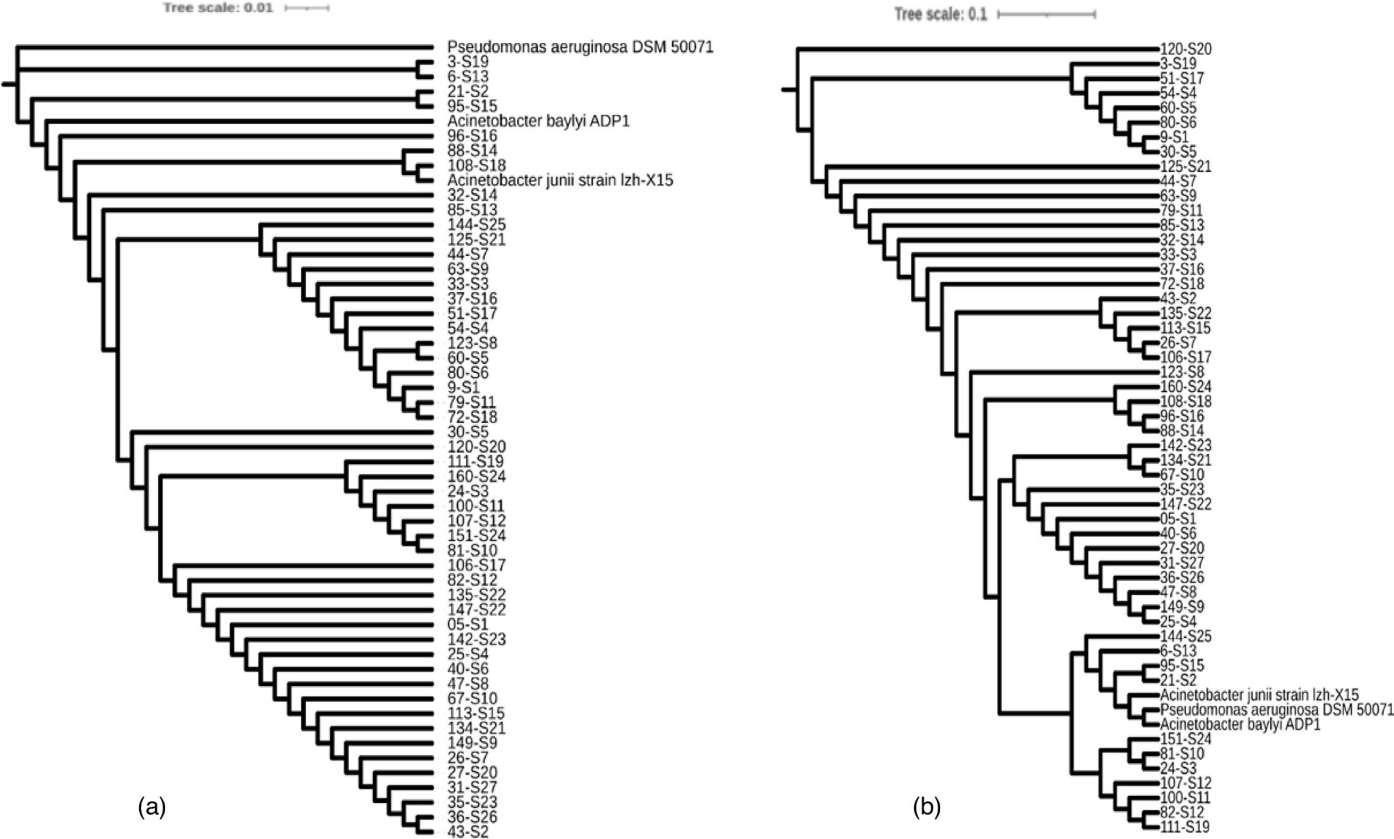
The phylogenetic tree of 51 strains of *A. baumannii* from information of 16S rRNA genes (a) and core genes (b) using the neighbour-joining method with three control species, including *A. baylyi* ADP1 (the same genus), *A. junii* lzh-X15 (the same genus) and *P. aeruginosa* DSM 50071 (the same order).

16S rRNA and Core genes of a total of 51 *A*. *baumannii* strain genes were sequenced by the MLST procedure. The obtained sequences were used to construct a phylogenetic tree utilizing specialized genetic analysis software (Fig. 1a, b). These findings serve as a foundation for further investigation into the epidemiological mapping of *A. baumannii* in Vietnam. Notably, the phylogenetic tree based on Core genes was superior to that of the 16S rRNA genes, as it successfully distinguished the three control species into a separate clade.

Table 1 shows the classification of the 48 *A*. *baumannii* strains into 11 STs. There were an additional three strains that remained unidentified. Notably, ST2 and ST571 exhibited the highest occurrence rates of 33.3 and 35.3%, respectively. This was followed by ST164 and ST575, both accounting for 5.9% of the strains. The remaining STs accounted for 1.96% of the strains. ST2 and ST571 exhibited the broadest distribution among the identified STs. Specifically, ST2 was found in four hospitals, including Thanh Nhan, Xanh Pon, Huu Nghi and 108 Military Central hospitals. Similarly, ST571 was detected in four hospitals, including Thanh Nhan, Xanh Pon, Huu Nghi and Can Tho hospitals. Notably, these STs were closely related to each other. Additionally, ST164 was found in three hospitals, namely Thanh Nhan, Hue and 108 Military Central hospitals. ST23 and ST16 were exclusively detected in 108 Military Central Hospital. On the other hand, ST109, ST52, ST1260, ST575, ST1223 and ST1336 were only detected in Thanh Nhan Hospital.

**Table 1.** Distribution of STs of 51 *A. baumannii* clinical strains

Hospitals	STs of 51 *A. baumannii* clinical strains											
	571	2	164	109	575	1260	1223	1336	52	16	23	–
Huu Nghi	2	4										1
Thanh Nhan	8	3	1	1	3	1	1	1	1			1
Hue			1									
Saint-Paul	5	8										1
Can Tho	3											
108 Military Central		2	1							1	1	
*n* (%)	18; 35.3	17; 33.3	3; 5.9	1; 1.96	3; 5.9	1; 1.96	1; 1.96	1; 1.96	1; 1.96	1; 1.96	1; 1.96	3; 5.9

Note: (–), unidentified.

### Analysing the relationship between STs isolated in Vietnam and the world

**Fig. 2.**
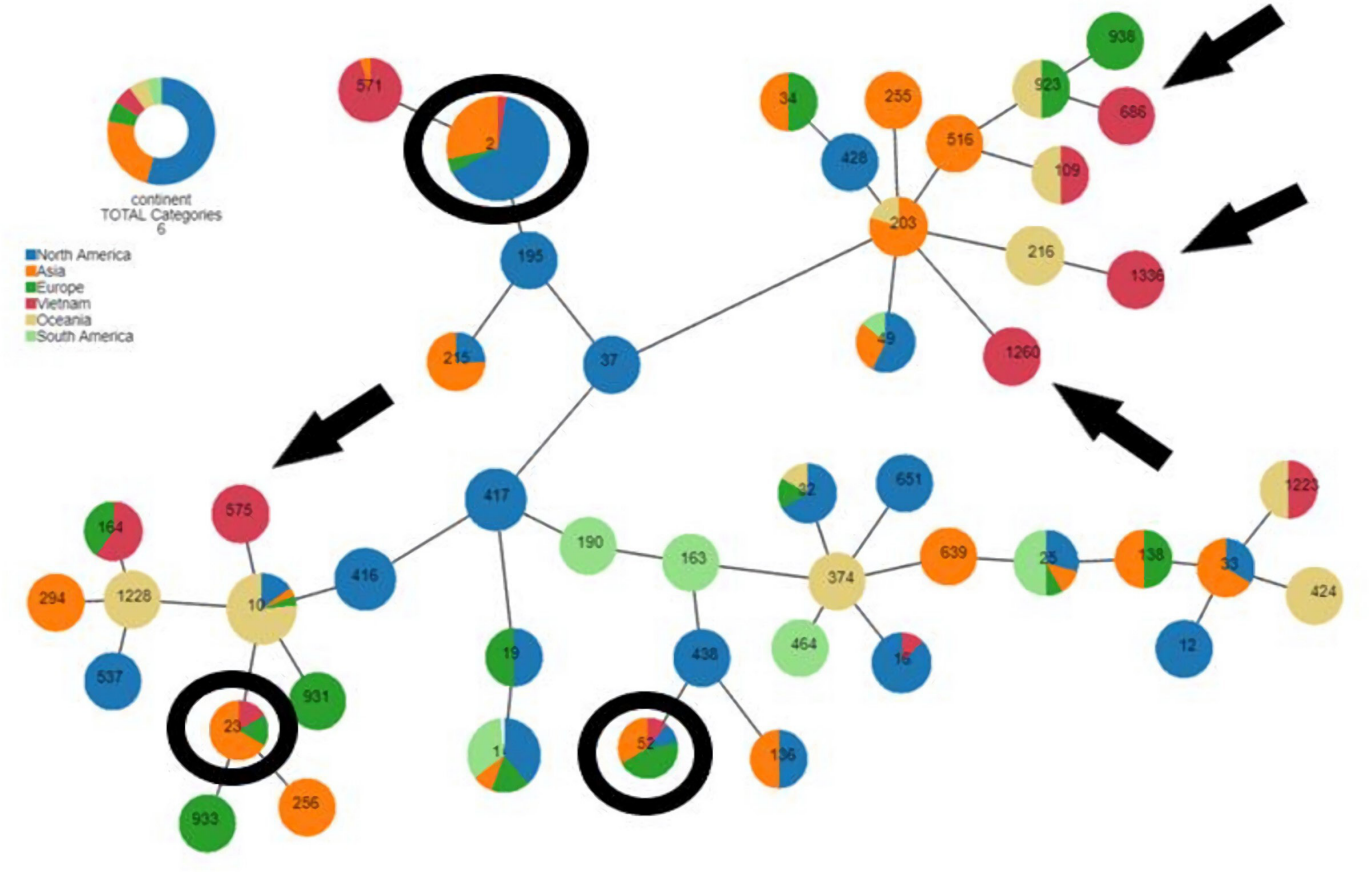
The diagram of STs isolated in Vietnam compared with those of different regions in the world belonging to the PubMLST database. The small circles with numbers represent the STs, while the colour on each circle indicates the occurrence of STs detected in different continents based on a specified colour code. Black circles indicate widely occurring STs worldwide. Black arrows are used to mark STs that were exclusively detected in Vietnam.

The relationship between STs of *A. baumannii* in Vietnam and worldwide data was determined by using the data from 48/51 ST-identified strains in this study and ST-identified strains that have been published on the PubMLST (https://pubmlst.org/). We selected STs related to the strains in Vietnam and common strains in the world. The relationship diagram was drawn by using the online software PhyLOViZ [42] (https://online.phyloviz.net/index); among them, each node represented distinctly an ST, and node size corresponded to the number of strains reported belonging to ST (the node size was normalized using logarithmic scale = 1). The results revealed that some STs were also found in Vietnam, including ST2, ST23 and ST52. Besides, STs were exclusively detected in Vietnam, such as ST686, ST1336, ST1260 and ST575 (Fig. 2). In general, strains with closely related genotypes belonged to the same ST or STs within the same clonal complex. This finding was expected, as STs were identified based on seven conserved housekeeping genes in the bacterial genome. The two most common STs found in this study were ST2 and ST571.

### AMR phenotyping of *A. baumannii* isolates

The results in Table 2 showed that *A. baumannii* was multiresistant to antibiotics: cefepime (48/51, 94.1%), ceftazidime (47/51, 92.2%), ciprofloxacin (46/51, 90.2%), levofloxacin (45/51, 88.3%), gentamicin (44/51, 86.3%), amikacin (37/51, 72.5%), imipenem (46/51, 90.2%), meropenem (45/51, 88.2%) and colistin (15/51, 29.4%). The results of MIC_50_ and of MIC_90_ were very high, such as cefepime (128 and >128 µg ml^-1^), ceftazidime (>128 and >128 µg ml^-1^), ciprofloxacin (32 and >32 µg ml^-1^), levofloxacin (>64 and >64 µg ml^-1^), gentamycin (>64 and >64 µg ml^-1^), amikacin (>256 and >256 µg ml^-1^), imipenem (64 and >64 µg ml^-1^), meropenem (32 and >64 µg ml^-1^), colistin (1 and 4 µg ml^-1^).

**Table 2.** Test results of MIC development of *A. baumannii* (*n* = 51)

Antibiotic group	Antibiotic	MIC rangeµg ml^−1^	MIC_50_ µg ml^−1^	MIC_90_ µg ml^−1^	*N* (%) (MIC µg ml^−1^)		
					*R*	*I*	*S*
Cephalosporin	FEP	2–>128	128	>128	48 (94.1) (32–>128)	0	3 (5.9) (2–8)
	CAZ	2–>128	>128	>128	47 (92.2) (64–>128)	0	4 (7.8) (2–8)
Fluoroquinolone	CIP	0.0625–>32	32	>32	45 (88.2) (16–>32)	1 (2.0) (2)	5 (9.8) (0.0625–0.5)
	LEV	0.06–>64	>64	>64	44 (86.3) (8–>64)	1 (2.0) (4)	6 (11.8) (0.06–1)
Aminoglycoside	GEN	0.125–>64	>64	>64	44 (86.3) (32–>64)	0	7 (13.7) (0.125–4)
	AMK	0.5–>256	>256	>256	35 (68.6) (≥256)	2 (3.9) (32)	14 (27.5) (0.5–16)
Carbapenem	IPM	0.125–>64	64	>64	45 (88.2) (8–>64)	1 (2.0) (4)	5 (9.8) (0.125–2)
	MEM	0.125–>64	32	>64	45 (88.2) (8–>64)	0	6 (11.8) (0.125–1)
Polymyxin	CL	0.125–≥4	1	4	15 (29.4) (4–8)	–	36 (70.6) (1–2)

FEP, cefepime; CAZ, ceftazidime; CIP, ciprofloxacin; LEV, levofloxacin; GEN, gentamicin, AMK, amikacin; IPM, imipenem; MEM, meropenem; CL, Colistin; *R*, completely resistant; *I*, intermediate resistant and *S*, sensitive.

### Antibiotic-resistant genes in 51 *A. baumannii* strains

**Fig. 3.**
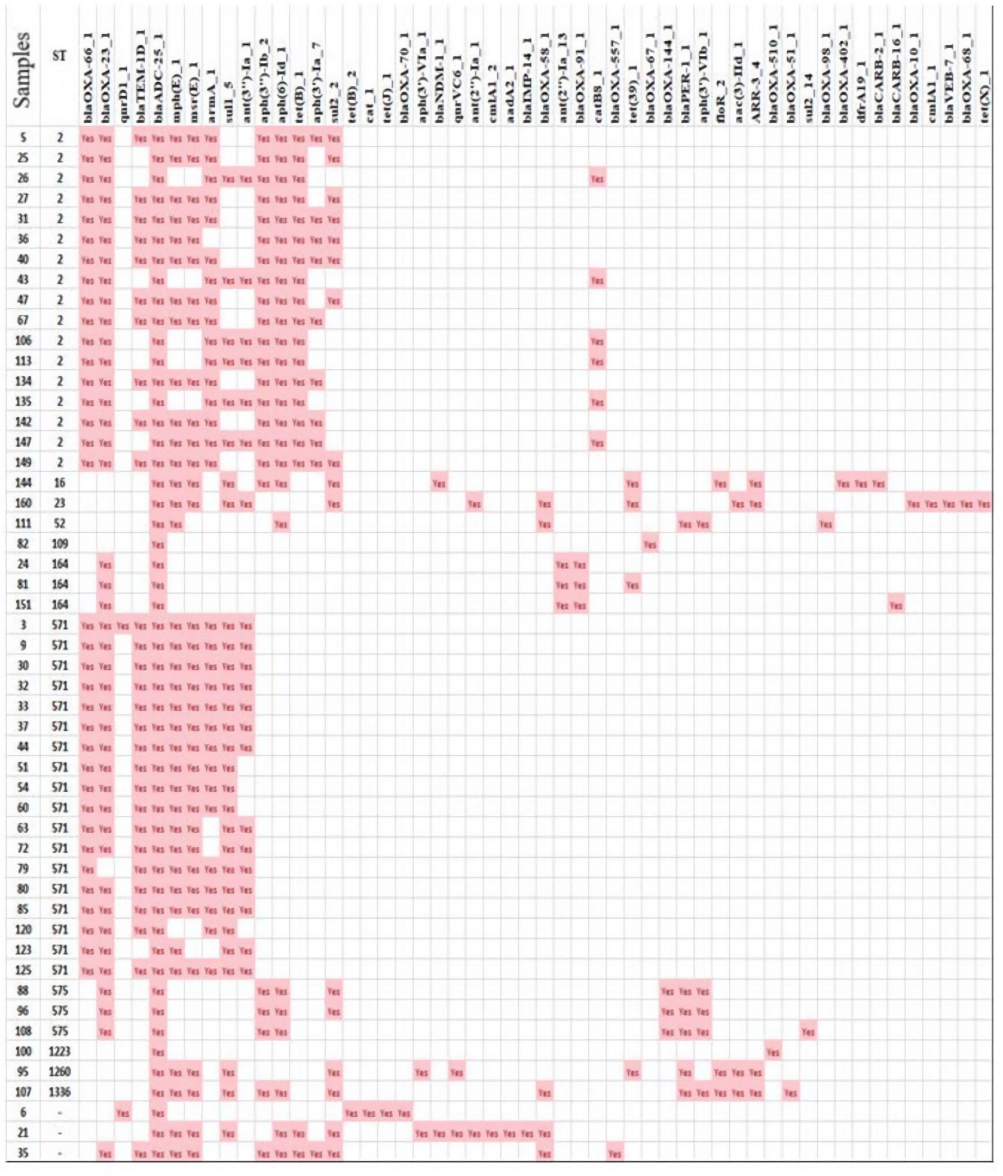
The relationship between STs and the occurrence of antibiotic-resistant genes.

**Fig. 4.**
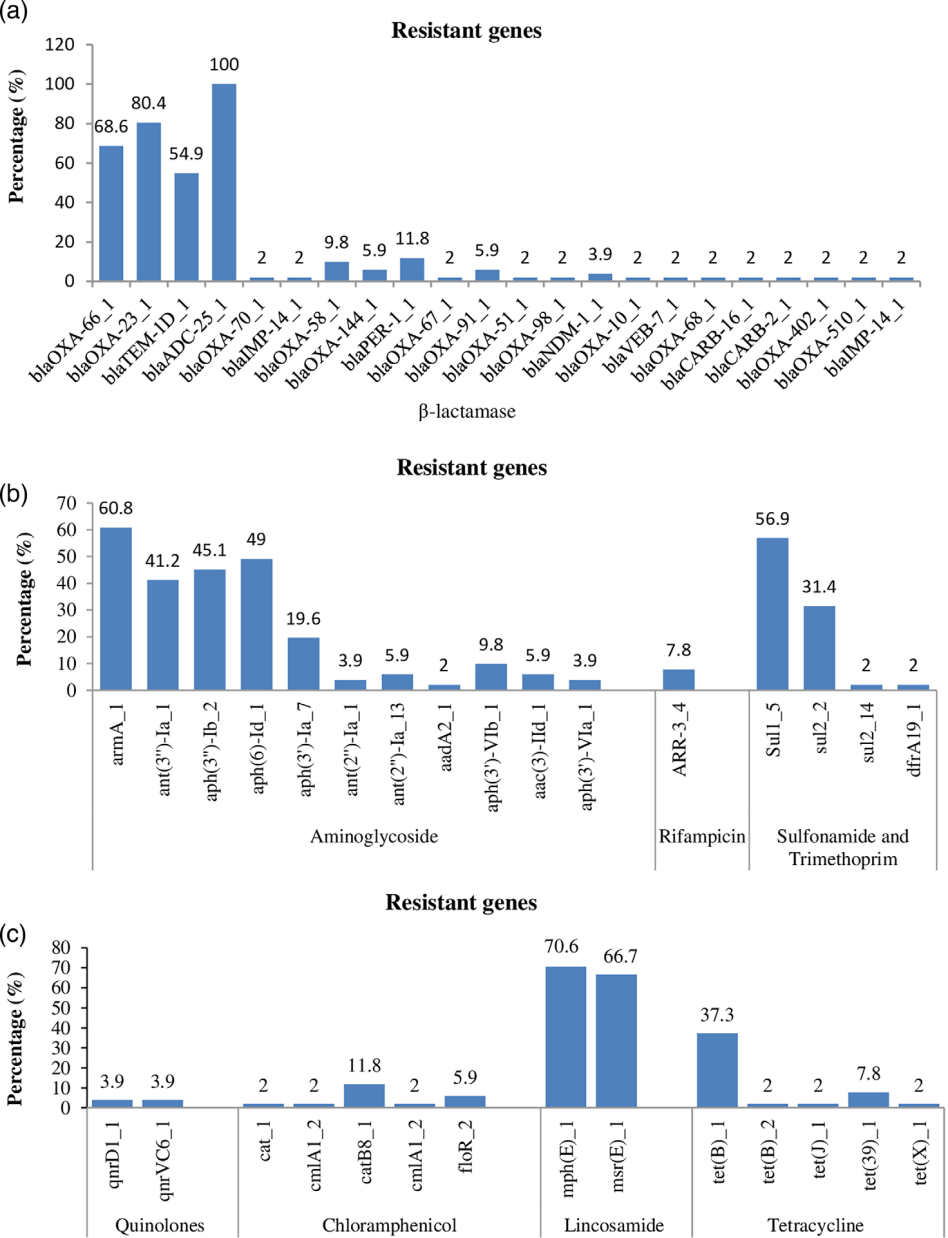
The prevalent of resistant genes: (a) resistant genes to β-lactamase; (b) resistant genes to aminoglycoside, rifampicin, sulfonamide and trimethoprim and (c) resistant genes to quinolone, chloramphenicol, lincosamide and tetracycline.

**Fig. 5.**
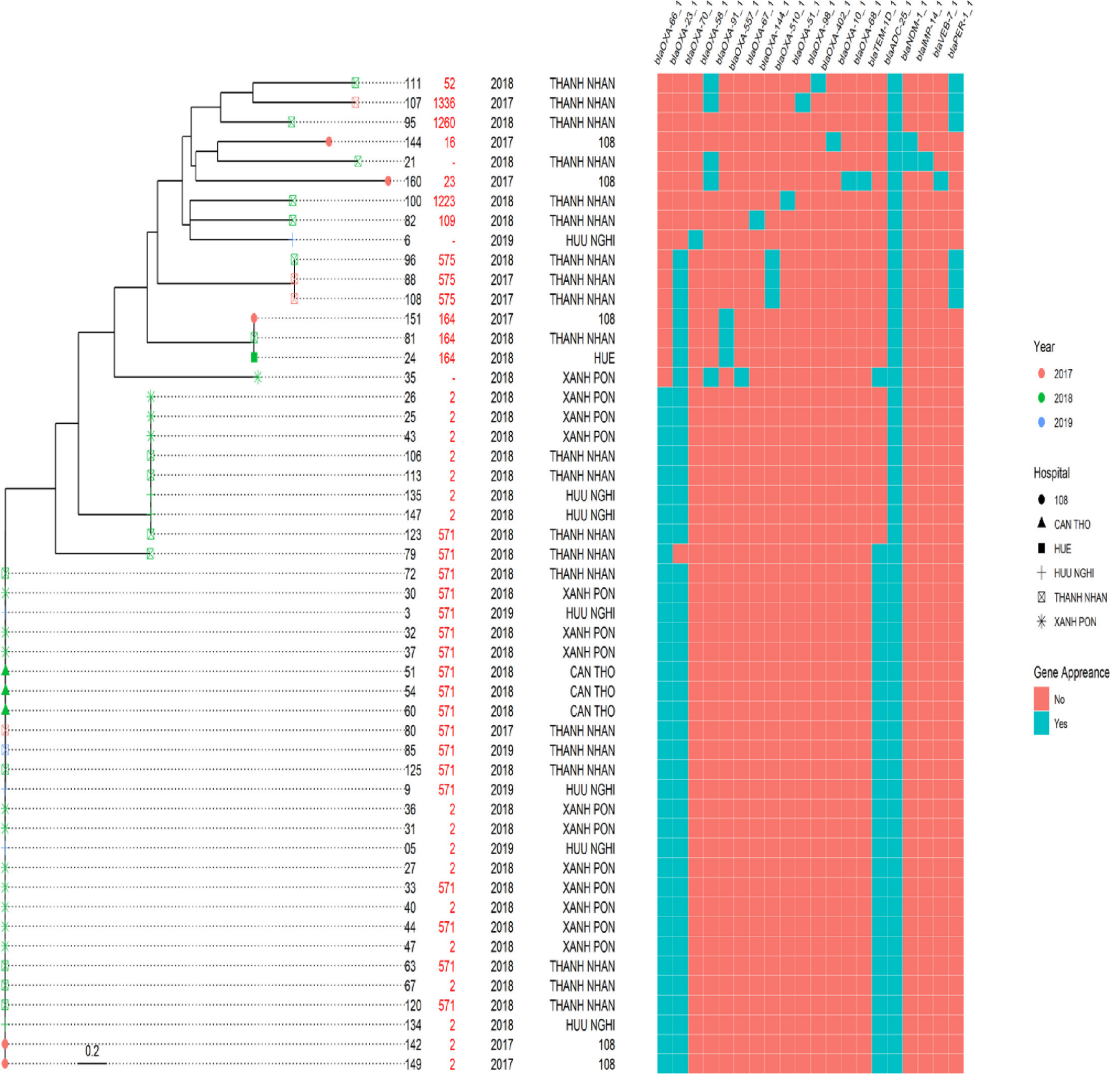
Relationships between strains were built on the presence of carbapenem-resistant genes.

The analysis revealed a total of 52 antibiotic-resistant genes in the genomes of 51 *A*. *baumannii* strains (Fig. 3). Among them, ST109 and ST1223 had the fewest number of resistant genes, with only two genes each. On the other hand, ST160 had the most resistant genes with 16 genes, followed by an unidentified ST (sample 21) with 15 genes. The prevalence of resistant genes for β-lactam, chloramphenicol, lincosamide, aminoglycoside, rifampicin, quinolone, sulfonamide, trimethoprim and tetracycline is shown in Fig. 4. Remarkably, the most common β-lactamase gene was *blaADC-25_1*, present in all 51 (100 %) strains. Additionally, *blaOXA-23_1* and *blaOXA-66_1* were found in 41 (80.4 %) and 35 (68.6 %) strains, respectively. The gene *blaTEM-1D_1* was detected in 28 (54.9 %) strains. Two strains (3.6%) carried the quinolone-resistant genes *qnrD1_1* and *qnrVC6_1*. For chloramphenicol resistance, the genes *catB8_1* and *floR_2* were found in 6/51 (11.8 %) and 3/51 (5.9 %) strains, respectively. Lincosamide-resistant genes *mph(E)_1* and *msr(E)_1* were detected in 36/51 (70.6 %) and 34/51 (66.7 %) strains, respectively. Tetracycline-resistant genes *tet(B)_1* and *tet (39)_1* were present in 19/51 (37.3 %) and 4/51 (7.8 %) strains, respectively. Aminoglycoside-resistant genes *armA_1*, ant (*3'')-Ia_1*, *aph (3'')-Ib_2*, *aph (6)-Id_1* and *aph (3')-Ia_7* were detected in 31/51 (60.8 %), 21/51 (41.2 %), 23/51 (55.1 %), 25/51 (49.0 %) and 10/51 (19.6 %) strains, respectively. The rifampicin-resistant gene *ARR-3_4* was found in 4/51 (7.8 %) strains. Sulfonamide-resistant genes *Sul1_5* and *sul2_2* were present in 29/51 (56.9%) and 16/51 (31.4 %) strains, respectively. We also investigated the correlation between the 51 strains and the 20 carbapenemase-encoding genes found among the 52 resistant genes (Fig. 5). The classification results demonstrated that strains with the same STs were clustered closely together on the phylogenetic tree. Therefore, it can be inferred that strains within the same ST possessed similar antibiotic-resistant genes, in which ST2 and ST571 stood out remarkably as they exhibited three genes encoding carbapenemase, namely *blaOXA-23*, *blaOXA-66* and *blaADC-25* (excluding sample 79). Additionally, 27/35 samples belonging to ST2 and ST571 carry the *blaTEM-1* gene.

### Mobile genetic structures carrying antibiotic-resistant genes

**Fig. 6.**
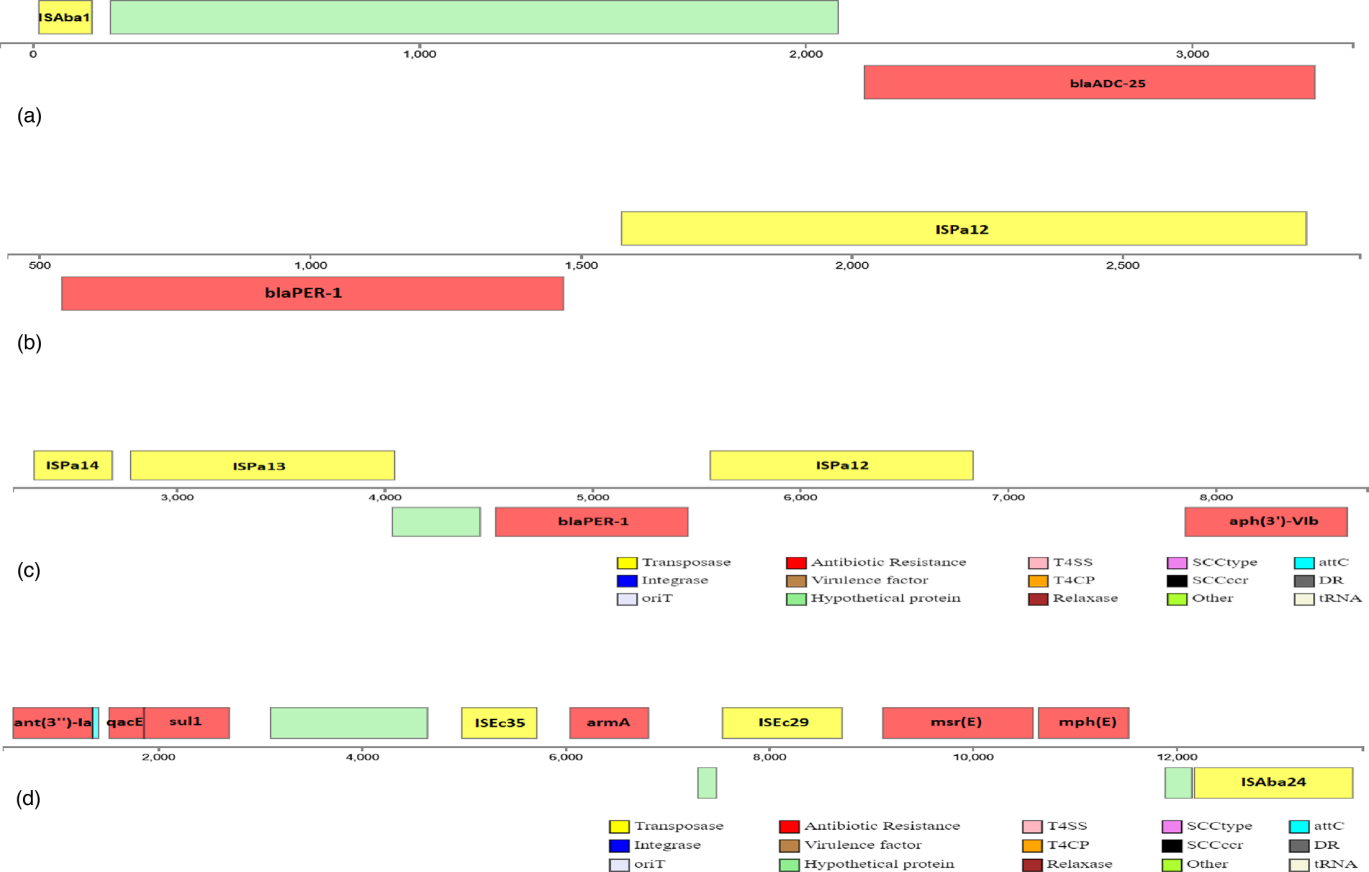
The transposon structure carrying the carbapenem-resistant gene in *A. baumannii* strains: 81 (a); 88 and 96 (b), 107 (c), and transposon structure ISEc35/ISEc29/ISAba24 (d).

The results showed that there are 21 insertion sequences/transposon clusters carrying different antibiotic-resistant genes (Table 3, Fig. 6). The most common of which is 11 ISEc35/ISEc29/ISAba24 transposon clusters carrying the *ant(3'')-Ia*/*qacE*/*sul1*/*armA*/*msr(E*)/*mph(E*) gene combination; next are six ISEc35 insertion sequences containing the *armA*/*sul1*/*qacE*/*ant(3'')-Ia*/*catB8* gene and five ISAba24/ISEc29/ISEc35 transposon clusters carrying the *mph(E*)/*msr(E*)/*armA* gene group.

**Table 3.** Genomic islands/transposons and AMR gene found in 51 strains

Genomic island/cluster transposon	Length (bp)	Antibiotic-resistant gene	Antibiotic group	Code
ISCfr1/ISAba14/ISAba14/ISAba14	6277	aac(3)-IId	Aminoglycoside	107
ISAbe12/IS1236/ISAba14/ISCfr1	6958	aac(3)-IId	Aminoglycoside	160
ISEc35/ISEc29/ISAba24	13 158	ant(3'')-Ia/qacE/sul1/armA/msr(E)/mph(E)	Aminoglycoside/disinfectant/sulphonamide/aminoglycoside/macrolide and streptogramin/macrolide	3, 9, 30, 32, 33, 37, 44, 54, 60, 67, 80, 85, 125, 134, 147
ISAba1/ISAba33	9514	aph(3'')-Ib/aph(6)-Id/tet(B)	Aminoglycoside /aminoglycoside/tetracycline	134
ISPa13/ISPa14	2703	aph(3')-VIb	Aminoglycoside	108, 111
ISEc35/ISEc29	3484	armA	Aminoglycoside	31, 51
ISEc35	6943	armA/sul1/qacE/ant(3'')-Ia/catB8	Aminoglycoside/sulphonamide/disinfectant/aminoglycoside/phenicol	26, 43, 63, 72, 79, 106, 113, 120, 135
ISAba1	3302	blaADC-25	β-Lactam	81
ISPa12	2298	blaPER-1	β-Lactam	88, 96
ISPa14/ISPa13/ISPa12	6319	blaPER-1/aph(3')-VIb	β-Lactam/aminoglycoside	107
ISAba34/ISAba2	2338	floR	Phenicol	107
ISAba2	4204	floR/sul2	Phenicol/sulphonamide	95
ISVsa3	6383	floR/sul2	Phenicol/sulphonamide	144
ISEc29	3989	mph(E)/msr(E)	Macrolide/macrolide and streptogramin	63
ISAba24	4618	mph(E)/msr(E)	Macrolide/macrolide and streptogramin	36, 79
ISAba24/ISEc29	6192	mph(E)/msr(E)	Macrolide/macrolide and streptogramin	72
Genomic island/ISAba24/ISEc29/ISEc35	9237	mph(E)/msr(E)/armA	Macrolide/macrolide and streptogramin/aminoglycoside	5, 25
ISAba24/ISEc29/ISEc35	8501	mph(E)/msr(E)/armA	Macrolide/macrolide and streptogramin/aminoglycoside	27, 40, 47, 142, 149
IS1006/ISAba33/ISVsa3	2746	sul2	Sulphonamide	107
ISCfr1	7356	tet(39)/mph(E)/msr(E)	Tetracycline/macrolide/macrolide and streptogramin	95
ISVsa5	3633	tet(B)	Tetracycline	6
ISAba1/ISAba33/ISMaq7/ISAba14/ISAba31	17 503	tet(X3)/sul2	Tetracycline/sulphonamide	160

## Discussion

*A. baumannii* is recognized as one of the most common nosocomial pathogens and an important reservoir of MDR genes. Our study was the first to investigate the epidemiology of *A. baumannii* isolated from patient specimens in the tertiary hospitals located across the North, Central and South regions of Vietnam. This is particularly significant given the limited data available on *A. baumannii* transmission in hospitals, especially in the intensive care units in Vietnam.

WGS of 51 *A*. *baumannii* strains, belonging to 11 STs, was performed to build the phylogenetic tree from two pieces of information of 16S rRNA and core genes (Fig. 1a, b). The results of the phylogenetic tree using the core genes provided better outcomes, as it successfully separated the three control species into a distinct clade. This separation is crucial for conducting more detailed analyses for epidemiological mapping purposes. The two most prevalent STs were ST2 and ST571, with incidence rates of 33.3 and 35.3%, respectively, with the widest distribution in four hospitals. These two STs were widely distributed across four hospitals and were found to be closely related to each other. Additionally, there were ST164 strains (5.9%) detected in three hospitals, and ST575 strains (5.9%) detected in one hospital. The remaining STs accounted for 1.96% of the strains and were isolated in only one hospital (Table 1). Our findings, along with some other studies, have shown that these isolated STs are widely distributed in all three regions of Vietnam, with ST2 and ST571 being the most dominant [43]. The prevalence of ST571 and, especially, ST2 in Vietnam is similar to that in other Asian countries, such as Thailand and Myanmar, where ST2 is considered responsible for the widespread and dominant spread of carbapenem-resistant *A. baumannii* strains [43].

Thus, *A. baumannii* strains carrying genes encoding carbapenemase in Vietnam are likely to have the same origin as the strains in Asia, specifically Southeast Asia. One of the reasons for this situation is the contiguous location and very high trade density between Vietnam and other countries in the region, such as Myanmar, Thailand, Singapore and Malaysia. The analysis results showed that these two STs had a close relationship, in which ST2 had the largest reported rate and the widest reported distribution in three regions of Asia, Europe and North America, in which the North American region accounted for the largest rate. ST571 had a smaller rate and is found mainly in Vietnam and some areas in Asia [43]. Chen et al. [44] investigated the strains isolated in Taiwan during 2015–2016 and 2018–2019, a country geographically close to Vietnam. This study identified STs such as ST455, ST787 and ST208 as predominant, with these types evolving over time in Taiwan [44]. Therefore, it is likely that *A. baumannii* strains carrying carbapenemase genes in Vietnam originated from the same source as strains in Asia, particularly Southeast Asia. One of the contributing factors to this situation is the geographical proximity and high volume of trade between Vietnam and neighbouring countries in the region, such as Myanmar, Thailand, Singapore and Malaysia.

Among the 51 strains analysed in this study, 48 strains were assigned to known STs, with 4 of them reported for the first time in Vietnam based on the PubMLST database. Furthermore, three strains were identified as belonging to new STs, which will be submitted to the PubMLST database for registration in the near future. In addition to the common STs found in many countries, the results also revealed the presence of ST1336, ST1260 and ST575, which were exclusive to Vietnam. These findings suggest that the emergence and spread of antibiotic-resistant *A. baumannii* strains in Vietnamese hospitals can be attributed to two main routes. First, it involves the spread of *A. baumannii* strains carrying resistance genes from other countries in the region and around the world. Second, it involves the development of antibiotic resistance under natural selection pressures resulting from the long-term and continuous use of antibiotics in clinical treatments. Regardless of the specific route, it is evident that the presence of antibiotic-resistant *A. baumannii* strains in Vietnamese hospitals has reached alarming levels. According to data from the PubMLST database, the most prevalent *A. baumannii* strains worldwide belong to ST2 and ST52, with wide distribution reported in Asia, Europe and North America. ST23 was reported in Asia and Europe, while ST164 and ST16 were isolated in Europe and America, respectively. ST109 and ST1223 were found in Oceania, while four other STs, including ST686, ST1336, ST1260 and ST575, were exclusively found in Vietnam. The presence of globally common strains in Vietnam serves as evidence of the global spread of these resistant strains through travel and globalization trends. Additionally, the identification of new STs exclusively in Vietnam provides evidence that strains in Vietnam have also developed in unique directions under the pressure of the widespread use of antibiotics in clinical treatments.

The rates of *A. baumannii* resistance to eight antibiotics belonging to four groups (except colistin) were very high (72.5–94.1 %) (Table 2), which is consistent with previous publications on the rate of dominant *A. baumannii* strains in Vietnamese hospitals [43] and some other countries [45,48]. Because of its resistance to multiple antibiotic classes worldwide, the increasing prevalence of MDR *A. baumannii* has led to limited therapeutic options. Besides, the rate of *A. baumannii* resistance to colistin (29.4%) is higher than that reported in some studies [45,47,49], but lower than in other studies [50,51]. It suggests that *A. baumannii* is beginning to develop resistance to colistin in Vietnam and other settings worldwide. This problem is becoming a public health concern as this antibiotic is used for the treatment of infections with carbapenem-resistant *A. baumannii*. In addition, the results of MIC_50_ and MIC_90_ were very high. Specifically, the MIC_50_ and MIC_90_ of amikacin, cefepime, ceftazidime, imipenem, meropenem, gentamycin and levofloxacin (Table 2). Our MIC results are similar to the rates in 14 Vietnamese provinces in the 2012–2013 study and many other international studies [52,55].

The analysis results revealed the presence of a total of 52 antibiotic-resistant genes in the genomes of the 51 *A*. *baumannii* strains (Fig. 4). Among them, ST109 and ST1223 had the fewest number of resistance genes, with only two genes each. On the other hand, ST160 and an unidentified ST (sample 21) had the highest number of resistance genes, with 16 and 15 genes, respectively. These resistance genes belonged to various classes, including β-lactam (22 genes), chloramphenicol (five genes), lincosamide (two genes), aminoglycoside (11 genes), rifampicin (one gene), quinolone (two genes), sulfonamides and trimethoprim (four genes) and tetracycline (five genes) (Fig. 4). Recent studies have also shown that *A. baumannii* strains exhibit abundant and diverse antibiotic-resistant genes across different regions [56,59]. This indicates that there is a wide range of antibiotic-resistant gene diversity in *A. baumannii* strains, which varies across different regions. This diversity can be attributed to the high level of intrinsic and acquired antibiotic resistance in *A. baumannii*, as well as its ability to readily spread within and between medical centres worldwide.

To further explore the relationships among the 51 strains, we constructed taxonomic trees using the presence of 20 carbapenemase-encoding genes, which are part of the 52 resistance genes (Fig. 5). The classification results demonstrated that strains belonging to the same ST were clustered together in the taxonomic diagram. Therefore, it can be concluded that strains with the same ST share similar antibiotic resistance genes. Specifically, ST2 and ST571 are characterized by the presence of three carbapenemase-encoding genes: *blaOXA-23*, *blaOXA-66* and *blaADC-25* (excluding unidentified sample 79). Additionally, 27 out of 35 isolated samples belonging to various STs carried the *blaTEM-1* gene. There are 21 IS/transposon clusters carrying different antibiotic-resistant genes. Construct ISAba1- blaADC (*A. baumannii* strain 81) and Construct ISpa12 were found to carry the *blaPER* resistance gene (*A. baumannii* strains 88 and 96). These findings have been found in many different regions worldwide [60,61]. Thus, the appearance of MDR strains with high genotypic similarities in Vietnam as well as from other countries in the region around the world shows the possibility of a strong spread of mobile genetic elements carrying resistance genes through recombination and horizontal gene transfer (Table 3, Fig. 6).

## Conclusion

The analysis of WGS data from 51 *A. baumannii* strains, collected from six hospitals in Vietnam, revealed that they belonged to 11 different STs. The genomes of these strains harboured a total of 52 resistance genes and were multi-resistant to antibiotics with very high MIC50 and MIC90. Overall, structural models of mobile genetic elements carrying antibiotic-resistant genes are quite diverse and have many similarities with countries in the region and around the world, showing the ability to rapidly spread drug-resistant strains through recombination and horizontal gene transfer. The results provided valuable insights into the distribution of different STs and identified key genetic determinants associated with antibiotic resistance in *A. baumannii* strains isolated from clinical settings in Vietnam.

## Abbreviations

*A. baumannii*: *Acinetobacter baumannii*
AMR: antimicrobial resistance
MDR: multidrug-resistant
MLST: multi-locus sequence typing
ST: sequence type
WGS: whole-genome sequencing
